# Stress, strain and deformation of poly-lactic acid filament deposited onto polyethylene terephthalate woven fabric through 3D printing process

**DOI:** 10.1038/s41598-019-50832-7

**Published:** 2019-10-04

**Authors:** Prisca Aude Eutionnat-Diffo, Yan Chen, Jinping Guan, Aurelie Cayla, Christine Campagne, Xianyi Zeng, Vincent Nierstrasz

**Affiliations:** 1 Textile Materials Technology, Department of Textile Technology, Faculty of Textiles, Engineering and Business, University of Borås, Borås, 50190 Sweden; 2 Laboratoire de Génie et Matériaux Textiles, ENSAIT/GEMTEX, Lille, 59000 France; 3 College of Textile and Clothing Engineering, Soochow University, Suzhou, 215006 Jiangsu China

**Keywords:** Polymer characterization, Mechanical properties

## Abstract

Although direct deposition of polymeric materials onto textiles through 3D printing is a great technique used more and more to develop smart textiles, one of the main challenges is to demonstrate equal or better mechanical resistance, durability and comfort than those of the textile substrates before deposition process. This article focuses on studying the impact of the textile properties and printing platform temperature on the tensile and deformations of non-conductive and conductive poly lactic acid (PLA) filaments deposited onto polyethylene terephthalate (PET) textiles through 3D printing process and optimizing them using theoretical and statistical models. The results demonstrate that the deposition process affects the tensile properties of the printed textile in comparison with the ones of the textiles. The stress and strain at rupture of the first 3D printed PLA layer deposited onto PET textile material reveal to be a combination of those of the printed layer and the PET fabric due to the lower flexibility and diffusion of the polymeric printed track through the textile fabric leading to a weak adhesion at the polymer/textile interface. Besides, printing platform temperature and textile properties influence the tensile and deformation properties of the 3D printed PLA on PET textile significantly. Both, the washing process and the incorporation of conductive fillers into the PLA do not affect the tensile properties of the extruded polymeric materials. The elastic, total and permanent deformations of the 3D-printed PLA on PET fabrics are lower than the ones of the fabric before polymer deposition which demonstrates a better dimensional stability, higher stiffness and lower flexibility of these materials.

## Introduction

3D printing technology using Fused Deposition Modeling (FDM) process has gained attention in the field of smart textiles due to its possibilities of integrating sensors, antennas and conductive tracks through deposition of layers of blended thermoplastic polymeric materials^1–5^. Smart textiles produced through deposition of filaments onto textiles using FDM process should demonstrate similar or better mechanical properties, durability and serviceability than the textile substrates to guarantee their use and development in textile industries. In this regard, the stress and strain at rupture and deformations under pressure of polymeric textile materials are relevant mechanical properties to study and enhance with both the process parameters and layers’ properties. The study of these mechanical properties can provide an in-depth understanding of the polymer/textile interface’s behavior.

Previously, researchers demonstrated that the mechanical properties of parts produced through Additive Manufacturing (AM) technique were mainly impacted by both the manufacturing process parameters and the physicochemical material properties before printing^6^. Mechanical anisotropy revealed to be the main issue of elements made using FDM process which was mainly caused by the shrinkage of the extruded thermoplastic deposited by the 3D printer, the layer-to-layer adhesion and the inconstant porosity when the printed track was in tension^7–12^. Most of the studies focused on tensile and yield strengths and elongation at break of the printed elements made of the most commercial filaments (Acrylonitrile butadiene styrene (ABS), Poly- lactic acid (PLA),..etc.)^13^. The layer thickness^14^, the orientation of the filament and the build platform^9,13,15^, the gap between the roads^9^, the printing trajectory^16^, the raster angle^9,13–15^ and other printing parameters^13,17–20^ have been considered as parameters which could influence the mechanical performance of the 3D-printed elements. Somireddy *et al*. also studied the influence of the layer thickness, road shape and air gap on the elastic moduli of a fused deposition processed layer^11^. In addition, the highest tensile strength of printed PLA sample was obtained when printing with 45° raster angle^13^. Tensile yield strength and tensile modulus of virgin PLA were measured and their values were respectively 40.3 MPa and 4258 MPa^7^. Besides, it could be demonstrated that the thinner and denser the layer of the 3D printed specimens, the higher the tensile strength^9,14^.

It has been widely publicized that polymeric blends or blended materials trend to reduce anisotropy^10,21–23^. Thus, in some cases, fillers or reinforcements are incorporated in polymers to enhance their mechanical properties^24^. Several studies have reported the mechanical properties of 3D printed nanocomposites^25–28^. The influence of the process parameters and textile characteristics on mechanical properties of printed textiles through other printing techniques such as digital printing was already investigated and revealed to be significant for some of the parameters such as fabric density and printing speed^29^.

Since 3D-printing process is performed on textile and not directly on a build platform, it is important to study the mechanical performance of 3D printed polymers on textiles materials and their interface, and also understand which textile substrates’ properties and process parameters influence their mechanical properties and durability the most. For instance, adhesion between the 3D printed polymers and the textile was previously studied. It was found that both 3D printing process parameters and textile properties could influence the adhesion property of these materials^1,2,4,5,30–35^.

Thus, this article focuses on studying the impact of the fabric’s properties and printing platform temperature on the stress, strain (SS-EN-ISO 13934- 1:2013) and deformations (using Universal Surface Tester (UST)) of non- conductive and conductive poly lactic acid (PLA) filaments (PLA + 2.5 wt%CB) deposited onto polyethylene terephthalate (PET) textiles through 3D printing process and optimizing them through theoretical and statistical models. In this study, the designation “3D-PPOT materials” is used to name 3D Printed Polymers On Textiles materials. In the case of use of conductive polymers, the appellation “3D-PPOT conductive materials” is preferred.

The findings of this study highly contribute in understanding the mechanical properties of 3D-PPOT materials produced through deposition process using 3D printing technology and used in the development of smart textiles^36–47^.

## Material and Methods

### Materials

The woven fabrics used were made of PET twisted multi-filaments of Nm 40, where Nm refers to the Number of hanks of 1000 meters/kg, as warp yarn and polyester monofilament of 0.2 mm in diameter as weft yarn. The non- conductive PLA monofilament (Ø = 1.75 mm) used for this 3D printing experiment was purchased at Creative tools AB. The extrusion process of the conductive nanocomposite PLA monofilaments was executed in a room, with a controlled temperature of 20 °C ± 0.2 and humidity of 65% ± 5. First, 2.5 wt. % of carbon black (CB) fillers (from Degussa, Evonik) were introduced into virgin PLA pellets (ref: 6202D from NatureWorks) and then dried in an oven set at 60 °C. Finally, the dispersion of the CB was executed using a Thermo Haake rotating and inter-meshing twin-screw extruder running at 100 rpm and at a range of temperature between 170 °C and 190 °C.

### 3D printing process

The 3D printing manufacturing process was done in climatized conditions (20 °C ± 0.2 as temperature and 65% ± 5 as humidity ratio). Polyester woven samples of rectangular shape (80 mm × 225 mm) were placed directly in the middle of a metallic build platform of the printer WANHAO Duplicator 4/4x prior to the printing process. Then, a thin and rectangular layer (50 mm × 200 mm × 0.1 mm) made of non-conductive or conductive PLA, designed first on Rhinoceros CAD software and then imported into Simplify 3D software, was printed on each different set of woven fabrics. The printing parameters are presented in Table 1. The distance between the head of the extruder and the surface of the textile was set during the calibration and remained constant and only the same extruder was used for all the different trials.

**Table 1:** Constant printing process parameters.

Parameters	Values
Infill percentage (%)	20
Z offset (mm)	0 mm (i.e. set distance between the nozzle and textile surface)
Printing speed (mm.min^−1^)	3600
Extruder diameter (mm)	0.4

### Dynamic surface deformations

Dynamic surface deformations, describing materials behavior and properties, were determined using a Universal Surface Tester (UST). Permanent and total deformations, elasticity, plasticity and viscoelastic properties could be obtained through this test. UST standard measurement consisted in variable load range scanning. During the measurement, a stylus was moving linearly on the sample surface for three times. The selected stylus had a diameter of 1.8 mm which is close to the one of the extruder head of the 3D printer. First, the stylus scanned continuously the material’s surface along a definite path with a minimum load of 1 mN. Then, the same path was scanned with the same stylus under a 60 mN constant load in order to simulate the applied pressure of the extruder head on textile substrate during 3D printing process. A deformation of the material’s surface occurred and was called total deformation. Third in this step, the same path was scanned again with the minimum load of 1 mN. The elastic part of the total deformation was recovered while the permanent deformation did not recuperate. Finally, the total, permanent and elastic deformations were calculated based on the differences between surface profiles of the replicates of the different steps. Five replicates were necessary to guarantee a good repeatability of the measurement. The dynamic surface deformations of the 3D-PPOT conductive and non-conductive samples were also measured, following the same steps previously explained, in order to compare them to the fabrics’ ones prior to printing process.

### Tensile and elongation at break properties

Tensile and elongation at break tests were carried out according to ISO 13934-1 standard. The speed was maintained at 100 mm/min and the length and width of the samples were 230 mm and 25 mm respectively. The distance between the two clamps was 100 mm. In this study, the tensile and elongation of both 3D- PPOT materials and the initial fabrics substrates were determined. The tensile and elongation at break values were an average of the three measurements. The maximum accepted standard deviation was 10%.

### Durability after washing process

The washing test was performed in a domestic washing machine following the standard SS-EN ISO 6330:2012. The washing process parameters are presented in Table 2. The washing process was applied on each sample separately. After the washing process, the tensile and elongation at break (stress and strain) measurements were performed on the 3D-PPOT non-conductive samples with 100% of PLA track bonded onto the textile substrates.

**Table 2:** Washing process parameters.

Detergent reference	ECE Formulation Non-Phosphate Reference detergent (A)
Detergent quantity	20 grs (±0.5) per liter of water
Complementary load fabric types	White cotton fabric – 950 grs per liter of water
Washing procedure	40 °C (±3) −15 mins including rinse and spin times (1 cycle)
Drying procedure	Open-air dry

### Statistical design of experiments

Four distinct factors (platform temperature, weft density as continuous factors, fabric orientation (weft and warp) and pattern as discontinuous ones) were defined and the order of the experiments were randomly created by Minitab 17 software. For each run, three replicates each were done. The values of the different factors used in the Design of Experiment (DoE) are presented in Table 3 for tensile test of the 3D-PPOT materials and deformation test of the textiles prior to printing process and Table 4 for deformation test of the 3D-PPOT materials. A full general factorial design was chosen for each experiment (i.e. all the samples were printed and tested) and analyzed through the “analyze factorial design” tool of Minitab 17. The statistical p-value and the contribution ratio are two important values that describe the significance of the defined factors’ impact on the measured responses, which are in our case the stress, strain and deformations of the 3D-PPOT materials. For a p-value below 0.05, the factor is considered as impacting and above 0.05 its influence is negligible. Besides, factors have different contributions that define their weight in the theoretical models. P-value and contribution are mainly used in this paper to study the impact of the different factors.

**Table 3:** Factors of statistical design of experiments for tensile measurements of the non- conductive and conductive 3D-PPOT materials and deformation of the textiles prior to printing process.

Factors	Values
Platform temperature	25, 60 and 100 °C
Weft density of the fabric	14, 18 and 22 picks/inch
Warp density of the fabric	20 picks/inch
Fabric direction	Machine (in warp yarn direction) and Cross (in weft yarn direction)
Fabric pattern	Plain or Twill 2/2 shown in Figure 1(a,b) respectively

**Table 4:** Factors of statistical design of experiments for deformation of the 3D-PPOT materials.

Factors	Values
Platform temperature	40 and 60 °C
Weft density of the fabric	14, 18 and 22 picks/inch Warp density of the fabric 20 picks/inch
Fabric pattern	Plain or Twill 2/2 shown in Figure 1(a,b) respectively

### Thickness measurement of fabrics

The thickness of each fabric was measured using a thickness gauge, micrometer KES-FB3 according to the standard ISO 5084. Since the fabric thickness is sensitive to pressure used during the measurement, an average of three measurements was necessary to guarantee satisfactory accuracy.

### Differential scanning calorimetry (DSC)

The 3D-PPOT using PLA filament printed on 14 picks/inch PET woven fabrics at three different temperatures 25, 60 and 100 °C in machine direction were used for DSC measurements in order to evaluate the influence of the platform temperature on their crystallization behavior and calculate the degree of crystallization. For DSC measurements, 6 mg samples were heated from 25 ^*°*^C to 260 ^*°*^C at 15^*◦*^/min in a nitrogen atmosphere. The formula used to calculate the degree of crystallization is presented in Eq. (1). The melting enthalpy of 100% crystalline PLA considered in the calculation is 93 J/g^48^.

1 $$degree\,of\,crystallization\,( \% )=\frac{Melting\,enthalpy\,(in\,J/g)}{Melting\,enthalpy\,(100 \% \,crystalline)(in\,J/g)}\times 100$$

## Results and Discussion

### Stress and strain of the 3D-PPOT materials through FDM process

#### Overall observation of the findings

Under a constant loading rate, the stress and strain of the 3D-PPOT materials made using FDM process were measured (Figure 2) and the raw data of the 14 picks/inch PET plain woven fabric printed with non-conductive and conductive PLA were presented in Figures 3–6. For both non-conductive and conductive 3D-PPOT materials, the PLA track was broken first at lower elongation range of [0.5%-3.5%] and lower tensile force range of [50N-250N] (Figs 3 and 4 (2)) following by the woven fabric at elongation range of [20%-35%] and tensile strength range of [300N-500N] (Figures 3 and 4 (3)). By comparing (1) and (3) of the stress-strain curves in Figures 3 and 4, it could be observed that the tensile strength at rupture of the woven fabric of the 3D-PPOT material was lower than the one prior to 3D printing. The stress and strain at rupture depend on the tensile properties of both layers (track and textile) as well as the adhesion resistance of their interface^1–5^. In the machine direction, the PLA track was broken at different points across the width (in the monofilament yarn’s direction) and then delaminates due to poor adhesion at the interface^5^. Whereas, in the cross direction, the adhesion at the interface is better and the PLA track was broken at one position without delamination (Figure 2).

**Figure 1:**
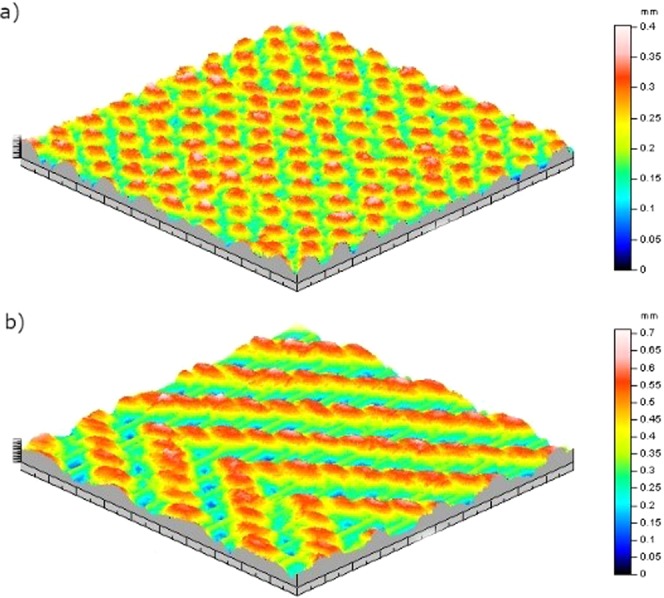
Plain (**a**) and Twill 2/2 (**b**) structures’ images obtained through profilometry technique.

**Figure 2:**
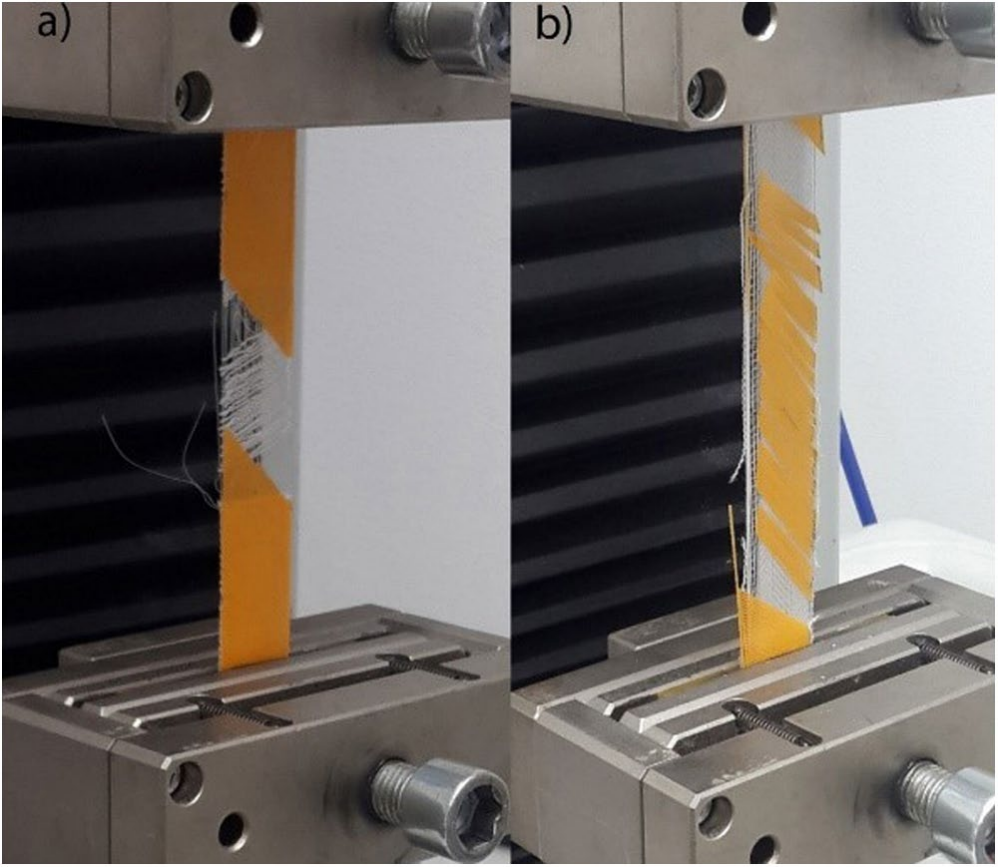
Strain and stress measurements of 3D-PPOT materials in cross direction (**a**) and machine direction (**b**).

**Figure 3:**
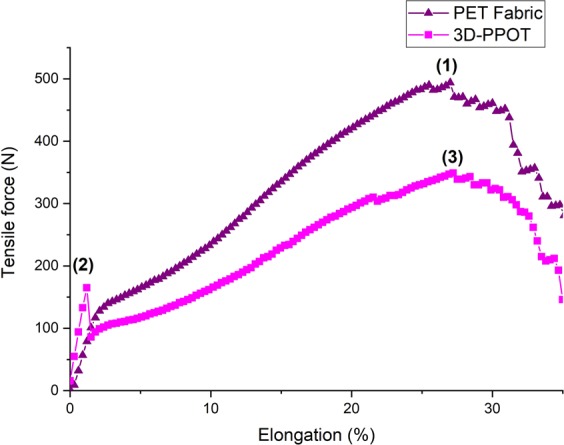
Tensile force–Elongation curves of 14 picks/inch PET plain woven fabric in cross direction and 3D-PPOT using PLA filament printed on 14picks/inch PET woven fabric at 25 °C in cross direction. (1), (2) and (3) represent the maximum strength of the 14 picks/inch PET plain woven fabric before printing, the maximum strength of the PLA/2.5%CB track of the 3D-PPOT and the maximum strength of the 14 picks/inch PET woven fabric of 3D-PPOT respectively.

**Figure 4:**
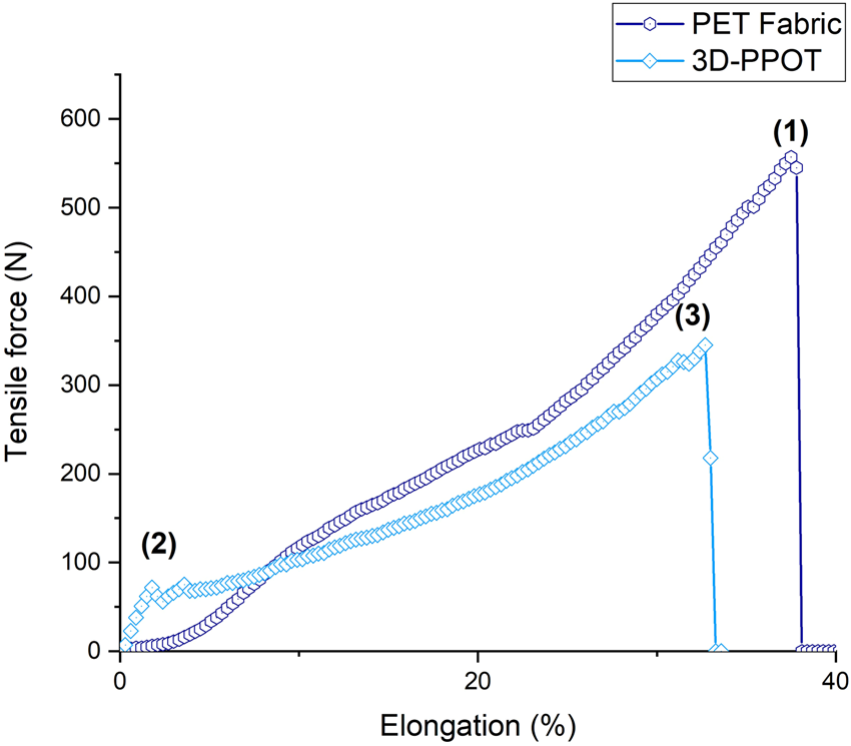
Tensile force–Elongation curves of 14 picks/inch PET plain woven fabric in machine direction and 3D-PPOT using PLA filament printed on 14picks/inch PET woven fabric at 25 °C in machine direction. (1), (2) and (3) represent the maximum strength of the 14 picks/inch PET plain woven fabric before printing, the maximum strength of the PLA/2.5%CB track of the 3D-PPOT and the maximum strength of the 14 picks/inch PET woven fabric of 3D-PPOT respectively.

**Figure 5:**
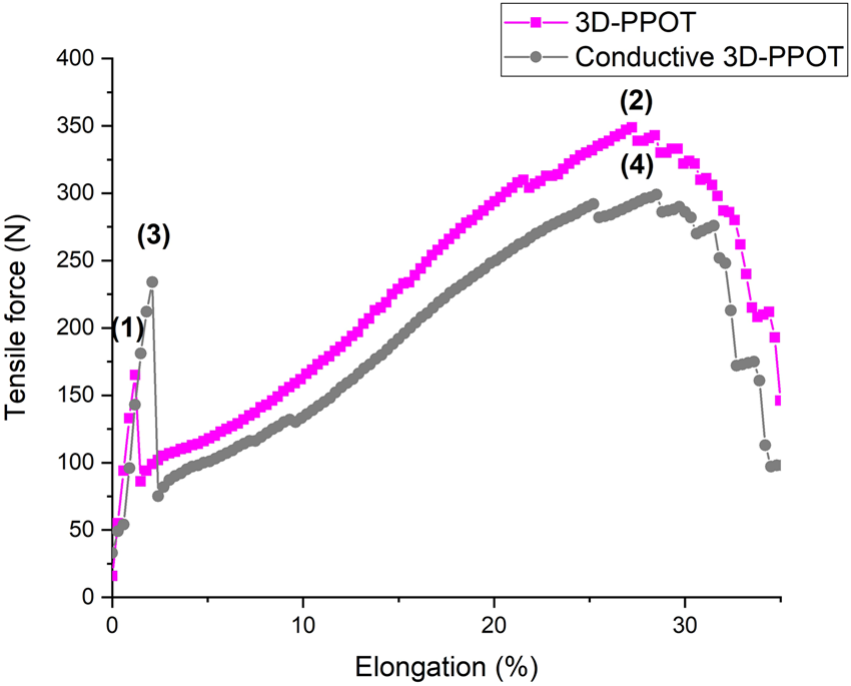
Tensile force–Elongation curves of 3D-PPOT materials using virgin PLA filament and PLA/2.5%CB (conductive) filament printed on 14 picks/inch PET woven fabrics in cross direction. (1) and (2) represent the maximum strength of the PLA layer and the PET fabric of the 3D-PPOT using virgin PLA filament printed in cross direction respectively. (3) and (4) represent the maximum strength of the conductive PLA layer and the PET fabric of the 3D-PPOT using PLA/2.5wt.% CB filament printed in cross direction respectively.

**Figure 6:**
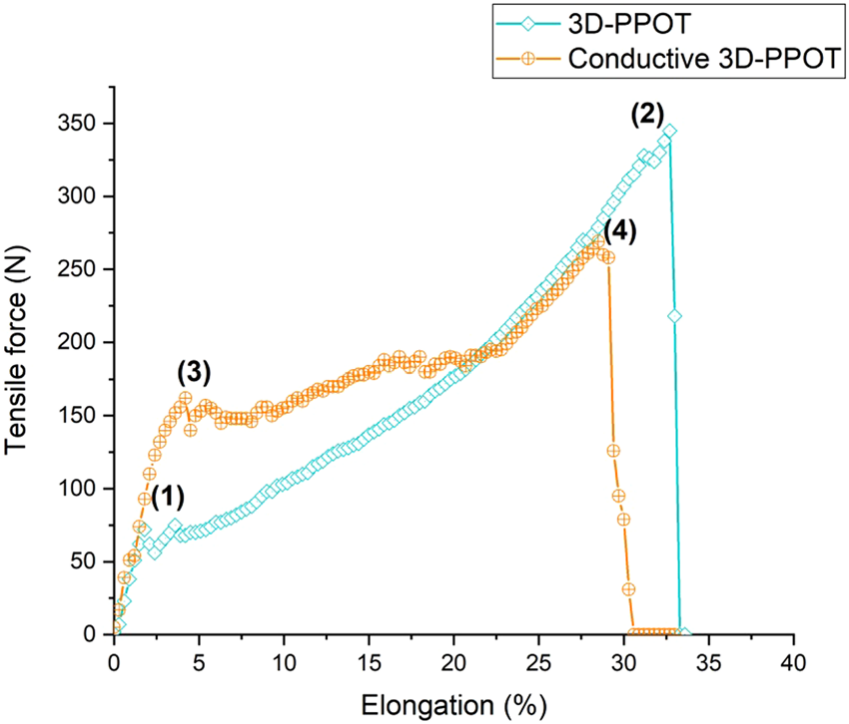
Tensile force–Elongation curves of 3D-PPOT materials using virgin PLA filament and PLA/2.5%CB (conductive) filament printed on 14 picks/inch PET woven fabrics in machine direction. (1) and (2) represent the maximum strength of the PLA layer and the PET fabric of the 3D-PPOT using virgin PLA filament printed in machine direction respectively. (3) and (4) represent the maximum strength of the conductive PLA layer and the PET fabric of the 3D-PPOT using PLA/2.5wt.% CB filament printed in machine direction respectively.

The overall stress and strain data were converted in megapascal following Eq. (2) and analyzed through Minitab 17 using DOE analysis tool.

2 $$Stress\,(MPa)=\frac{tensile\,force\,(N)}{25\times t}$$

where t is the thickness of the material (in mm).

**Figure 7:**
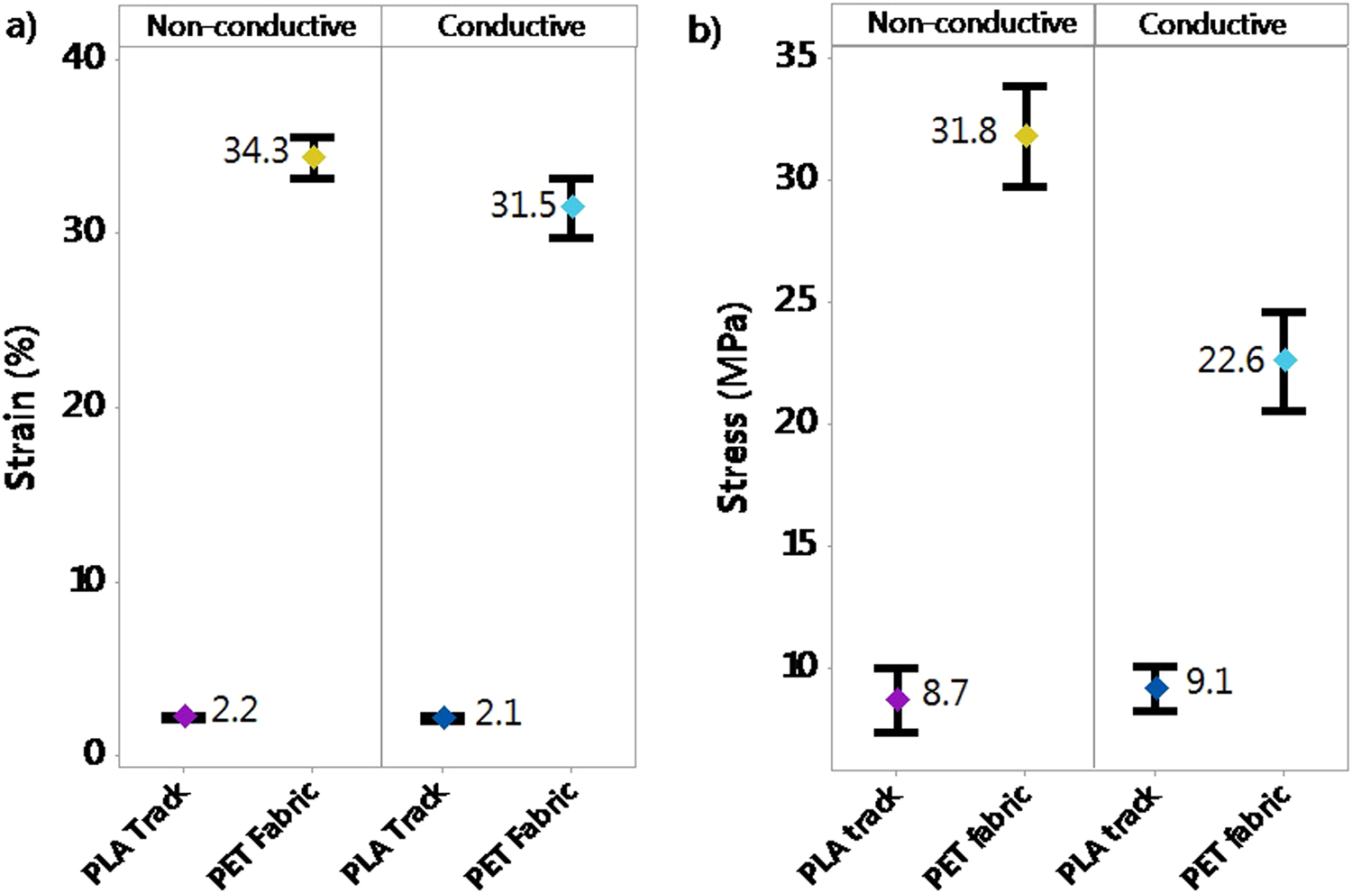
Strain in % (**a**) and stress in MPa (**b**) and at rupture of the non-conductive and conductive PLA track printed deposited onto PET fabric (3D-PPOT) composed of both stress and strain at rupture of PLA track and PET Fabric.

An in-depth analysis of all data of the DoE confirmed that 3D-PPOT materials’ stress and strain were a combination of the ones of the PET fabrics and the PLA printed track. With mean values of 34.5 MPa and 34.3% for the fabric and 8.7 MPa and 2.2% for non-conductive PLA layers, the stress and strain of the fabric remained the highest (Figure 7). The same trend could be highlighted in the case of use of conductive PLA onto PET fabric. The PLA printed layer was not broken in the same manner when deposited in machine or cross direction of the polyester textile substrate.

#### Influence of conductive fillers incorporation

By considering the stress values of the entire DoE using both PLA/2.5%CB and pure PLA filaments of tensile strength values of 11890 cN/Tex (1Tex equals 1 gram per 1000 meters) and 11659 cN/tex respectively, no significant difference was observed in the mean values and interval of the stress and strain of both pure PLA (8.7 MPa and 2.2%) and PLA/2.5%CB (9.1 MPa and 2.1%) PLA filaments (Figure 7), i.e. the percentage of carbon black filler introduced (2.5 wt. %) into the neat polymer did not significantly affect the tensile proper- ties of the 3D-PPOT materials using FDM process. However, the stress-strain curves of the 14 picks/inch plain woven fabric demonstrated that the addition of CB fillers enhanced considerably the stress of the PLA track (Figures 5 and 6). The findings were in consistency with the ones of Kausar *et al*. who showed that the addition of nanomaterials into polymers for 3D printing use often reinforce the composite polymers and improve the mechanical properties of the printed composites parts^49^. By adding 10 wt. % carbon nanofiber^50^ or 10 wt. % multi-walled carbon nanotubes (MWCNT)^51^ the tensile strength of the printed parts raised by 39% and 7.5% respectively and reduced the elongation. Thus, higher weight percentage of carbon black could be incorporated in order to increase the tensile strength of the textile while not reducing its strain. Besides, it could be observed that the stress of the fabric is reduced by approximately 3% when printing with conductive PLA and the strain is not affected (Figure 7).

#### Effect of textile properties and printing platform temperature on the stress and strain of the 3D-PPOT materials

The build platform (or printing platform) temperature of the 3D printer as well as the weft density, pattern and direction of the fabric have a significant impact (Tables 5–7) on the stress of the 3D-PPOT materials, i.e. the textile and printed PLA layer, but no impact on their strain. The direction of the fabric and the platform temperature revealed to be the factors with the highest and the lowest contributions in the non-conductive and conductive PLA stresses respectively. The pattern had a higher impact on the stress of the conductive PLA of 3D-PPOT materials compared to the stress of the non-conductive PLA (Tables 5 and 7). The weft density and direction are the factors with the highest contributions in the PET woven fabric stress, i.e. in proportion they have a higher effect on those responses.

**Table 5:** Stress at rupture of non-conductive of PLA track of 3D-PPOT material (MPa): p-values and contributions of the main factors.

Factors	P-values^a^	Contribution^b^ (%)
Platform temperature (°C)	0.00	11.42
Weft density (pick/cm)	0.00	3.87
Pattern	0.00	0.66
Direction	0.00	65.66

^a^Determine statistical significance of factors’ effect on non-conductive PLA stress.

^b^Describes the weight of each main factor in the statistical model.

**Table 6:** Stress at rupture of PET woven fabric of 3D-PPOT material (MPa): p-values and contributions of the main factors.

Factors	P-values	Contribution (%)
Platform temperature (°C)	0.00	1.29
Weft density (pick/cm)	0.00	19.57
Pattern	0.00	0.02
Direction	0.00	53.50

**Table 7:** Stress at rupture of conductive PLA track of 3D-PPOT conductive material (MPa): p-values and contributions of the main factors.

Factors	P-values	Contribution (%)
Platform temperature (°C)	0.00	11.15
Weft density (pick/cm)	0.00	0.51
Pattern	0.00	12.93
Direction	0.00	53.40

**Figure 8:**
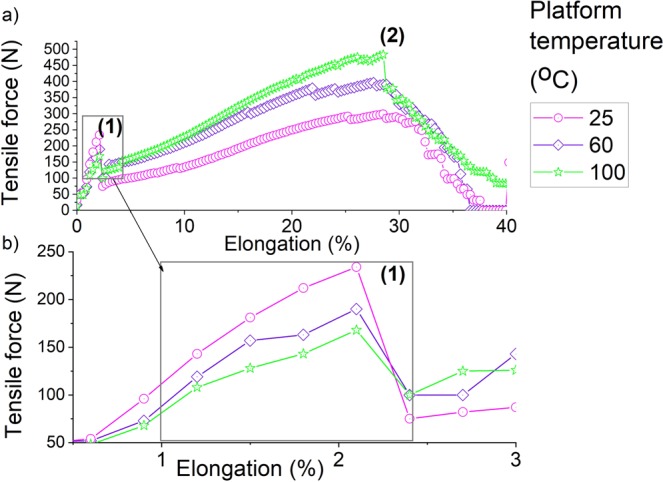
Tensile force–Elongation curves of 3D-PPOT using PLA filament printed on 14 picks/inch PET woven fabrics at three different temperatures 25, 60 and 100 °C in machine direction (**a**). (**b**) is a focus image of (**a**). (1) and (2) represent the maximum strength of the PLA layer and the PET fabric of the 3D-PPOT using virgin PLA filament respectively.

**Figure 9:**
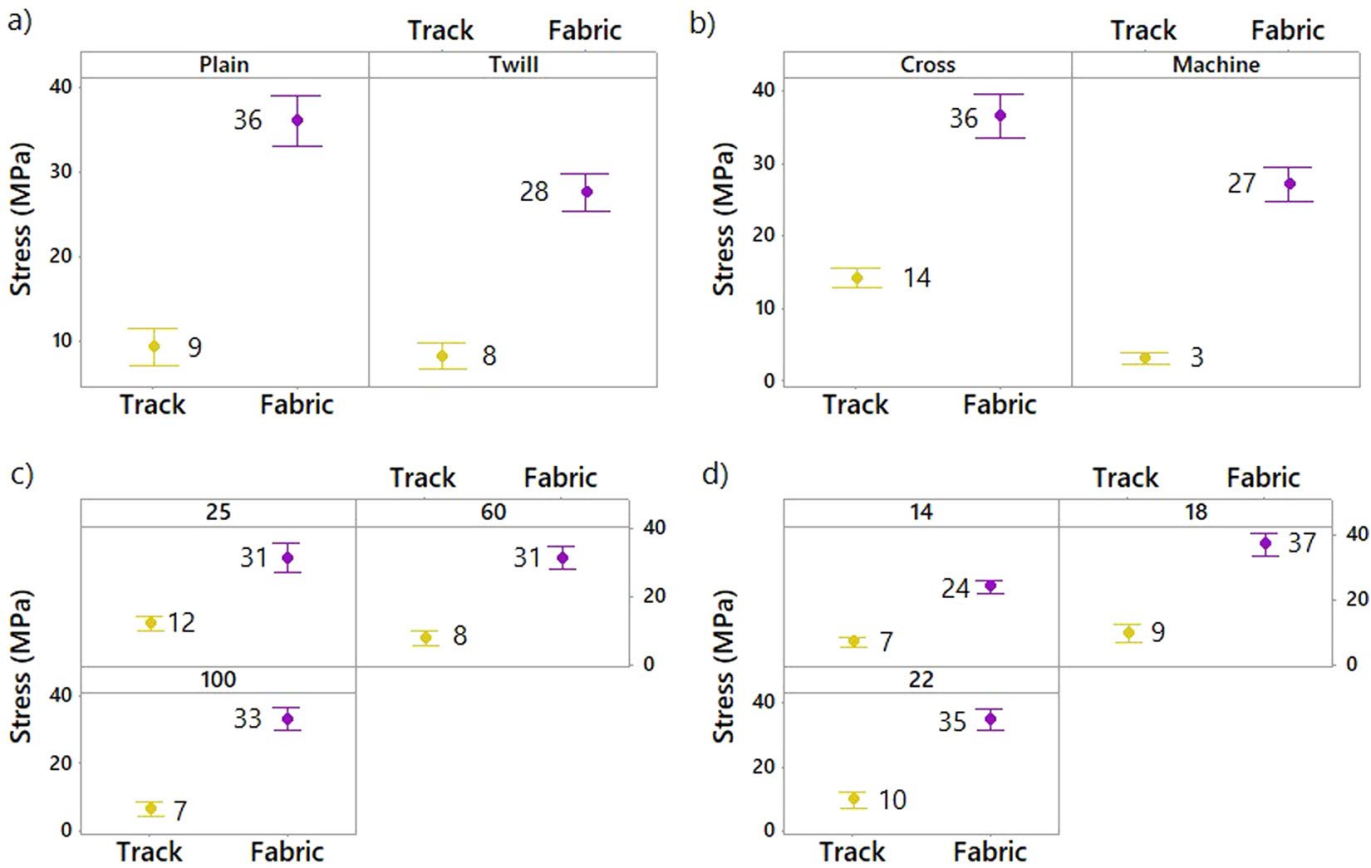
Effect of textile pattern (**a**), textile orientation (**b**), platform temperature (**c**) and textile weft density (**d**) on stress (MPa) of 3D-PPOT materials made of non-conductive PLA track and PET fabric. Both the stress of the PLA track and PET fabric have to be considered.

**Figure 10:**
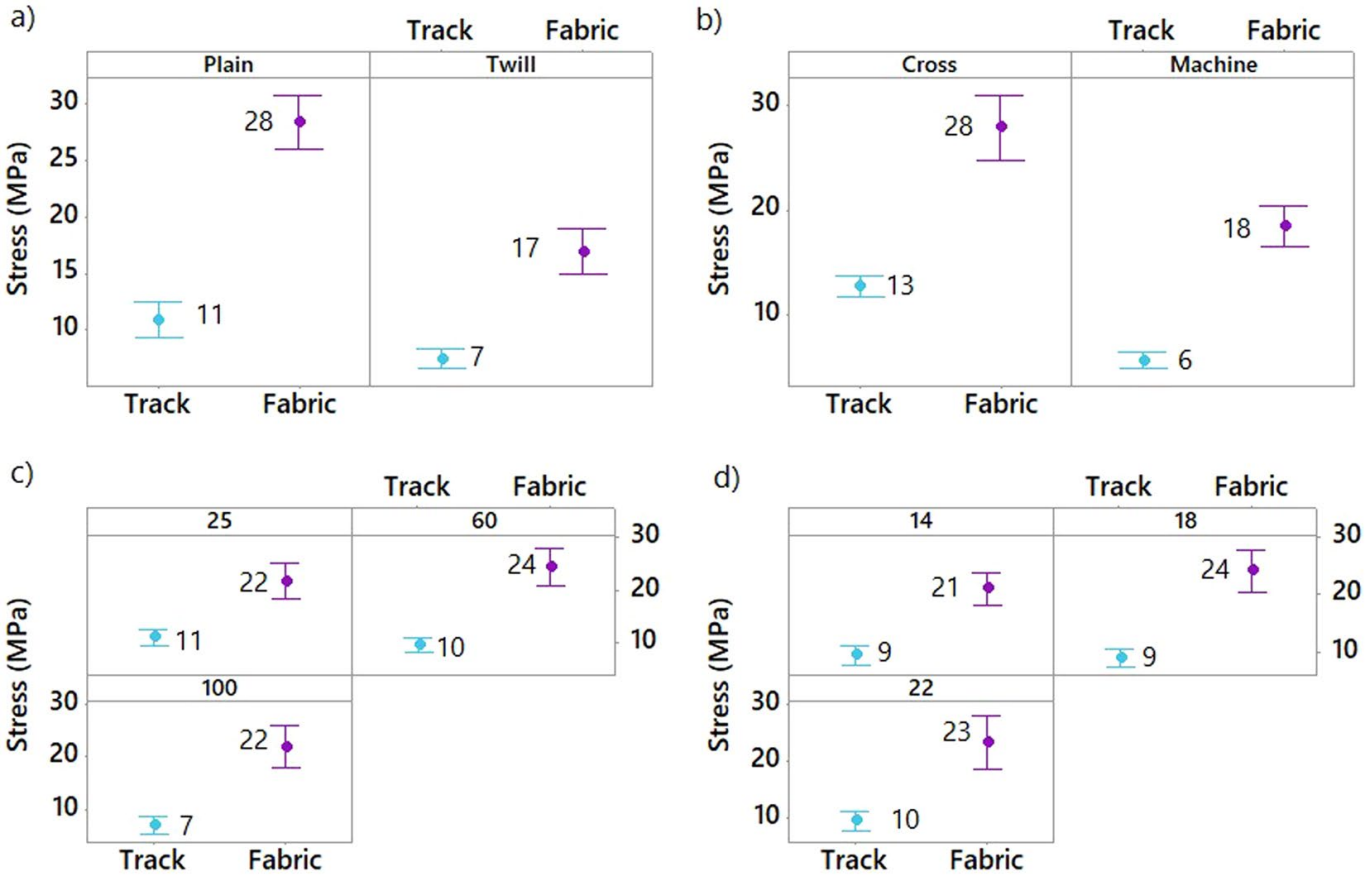
Effect of textile pattern (**a**), textile orientation (**b**), platform temperature (**c**) and textile weft density (**d**) on stress (MPa) of conductive 3D-PPOT materials made of conductive PLA track and PET fabric. Both the stresses of the PLA track and PET fabric have to be considered.

**Figure 11:**
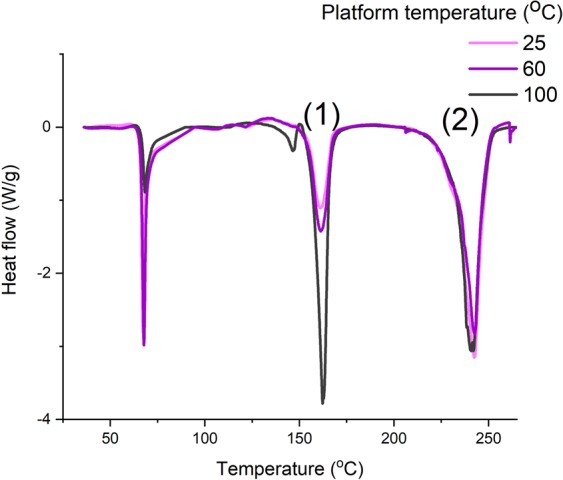
DSC curves of conductive 3D-PPOT materials when using 25, 60 and 100 °C as platform temperature. (1) is the melting peak of PLA and (2) is the melting peak of PET.

The effect of the pattern, the fabric orientation (cross or machine), the platform temperature and the weft density of the woven material on the stress of the non-conductive and conductive 3D-PPOT (track and textile) was shown in Figures 8–10. The stress of the non-conductive and conductive PLA track is higher in the cross direction than in the machine direction (Figures 9 and 10). The lower the platform temperature and higher the weft density, the better the stress of the non-conductive PLA track (Figures 8 and 9). Similar trends can be observed with the conductive PLA filaments (Figure 10). Taubner and Shishoo studied the processing temperature during extrusion process of the PLA polymer and its effect on the average number molecular weight and found higher polymer degradation by increasing temperature^52^. Also, it has been already demonstrated that PLA and more specifically poly-L-lactide (PLLA) presented ester linkages which trended to degrade under high temperature conditions. In the present study, an increase of the platform temperature up to 100 °C have led to increase the degree of crystallinity of PLA polymer, as presented in Figure 11 and Table 8, due to a decrease of its crystallization kinetics (speed for instance). The PLA crystallinity degree was calculated with a melting enthalpy of 100% crystalline of 93 J/g^48^. In previous studies^53,54^, the temperature was already found to be a critical parameter which affected the crystallization behavior of polymers. An increase of temperature might trend to enhance of chain mobility that controls the crystallization capacity of polymer. Also, the cooling process of the polymer might have occurred more rapidly and affected the re-arrangement of the molecular chains of the polymer by creating less ordered structure (e.g., crystal modification and degree of crystallization)^55^. Therefore, with a glass transition temperature in the range of 50–60 °C, the PLA polymer is more rigid and brittle below this range of temperature and thus, demonstrated lower stress at high platform temperature.

**Table 8:** DSC characterization of conductive PLA of 3D-PPOT materials when using 25, 60 and 100 °C as platform temperature during 3D printing process.

Platform temperature (°C)	PLA Melting temperature (°C)	PLA Melting enthalpy (J/g)	Crystallinity Degree of PLA (%)
25	160	4.6	5.0
60	161	6.5	7.0
100	162	13.5	14.5

Besides, it has been already proven that the adhesion strength of the conductive and non-conductive 3D-PPOT materials is higher with an increase of the platform temperature above the glass transition temperature of the polymer^5,31,56^. Therefore, at 100 °C, the polymer can remain longer at molten stage allowing a stronger penetration of the polymer through the textile structure and better anchor. A better adhesion of 3D-PPOT material could explain the lower stress of the PLA track due to its lower thickness and much higher affinity with the PET fabric.

Furthermore, Ma *et al*. demonstrated that the interfacial debonding was the main failure mode of ultimate tensile of twisted sisal yarns reinforced composites whereas the failure mode for non-twisted sisal yarns reinforced composites was the yarn breakage^57^. In our case, woven fabrics used were made of PET twisted multi-filaments of Nm 40, where Nm refers to the Number of hanks of 1000 meters/kg, as warp yarn and polyester monofilament of 0.2 mm in diameter as weft yarn [section 2.1]. Thus, the PLA monofilament might have higher affinity with the PET monofilament compared to the twisted PET multi-filaments, resulting in different cracks and a debonding of the PLA layer in the machine direction during tensile test. The Van-der-Waals forces might be stronger between the layers after printing in the cross direction than those after printing in the machine. Similar trends of results obtained for stress and strain measurements were achieved when testing the adhesion of the same samples and better adhesion seems to lead to better tensile properties^5^.

Before 3D printing process, the four factors had significant influence on the stress of the fabric either printing with non-conductive or conductive PLA filament (p < 0.05). It was revealed that plain fabric had higher tensile strength than twill one. This trend, even unexpected due to the high crimp of plain leading to lower mechanical properties^58^ was fully reported by researchers who investigated the influence of textile properties on the tensile strength of textile^57,59,60^. After 3D printing process, the non-conductive and conductive 3D-PPOT materials have significantly higher stress at rupture when printing in the cross direction, due to more resistant monofilament used as weft yarn and increasing the weft density leading to a closer packing of the structure.

### Permanent, elastic and total deformations of the 3D-PPOT materials

#### Overall observation of the findings

**Figure 12:**
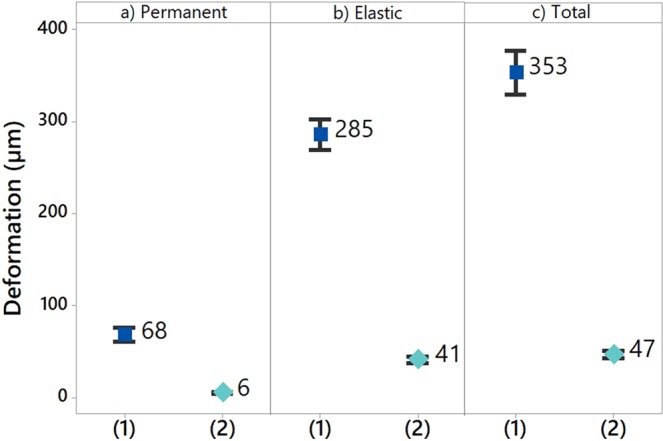
Permanent (**a**), elastic (**b**) and total (**c**) deformations before (1) and after (2) deposition process through 3D printing.

The permanent, elastic and total deformations of the textile materials before and after FDM process were measured and compared in order to understand the effect of the deposition process on the change in mechanical properties of the 3D-PPOT materials in comparison to the one the fabric used as a substrate. With permanent, elastic and total deformations decreases of 88%, 85% and 87% respectively, it was demonstrated that the virgin PLA deposition created stiffer and more stable fabric (Figure 12). Indeed, the deformations of materials were lower due to the high young’s modulus of the processed PLA filament (~3.5 GPa) and higher crystallization rate which created stiffer materials. Besides, the deposition process reduces the pores of the fabrics and thus its flexibility and drapability. It also improved the dimensional stability of the textile and its comfort^61^.

#### Effect of textile properties and printing platform temperature on the deformation of the 3D-PPOT materials

**Figure 13:**
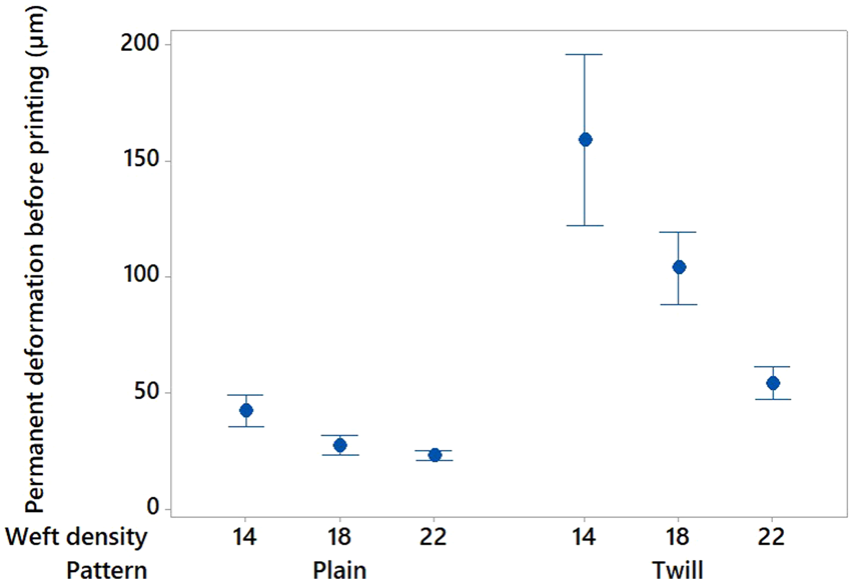
Main effects plot: permanent deformation (in µm) of fabrics before printing versus weft density (picks/inch) and pattern.

**Figure 14:**
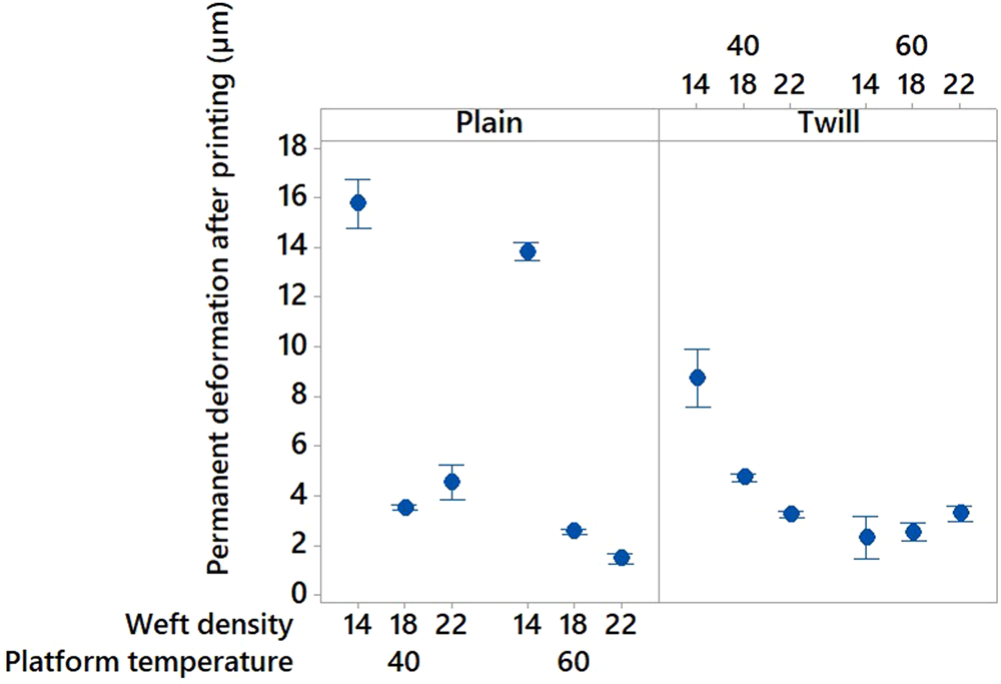
Main effects plot: permanent deformation (in µm) of the 3D-PPOT materials versus weft density (picks/inch), pattern and platform temperature (°C).

Before printing, the pattern and the density of the textile materials revealed to have noteworthy impact on its deformation. Indeed, twill pattern demonstrated to be more flexible than plain pattern with higher permanent (Figure 13), elastic and total deformations; and increasing the weft density of the fabric decreased its flexibility (Figure 13). These observations can be explained by the yarns arrangement within the two different textile structures. In the plain weave, each weft thread crosses the warp one by going simultaneously over and under whereas in the twill 2/2 the weft thread goes simultaneously over two warp threads and then under two. Consequently, twill 2/2 weave fabrics present floats which deliver more streachibility to the fabrics and higher elastic permanent and total deformation. After printing, the pattern, weft density and platform temperature impacted significantly the permanent deformation (p-value = 0 in Table 9, Figure14) however, the elastic and total deformations were only affected by the weft density (p-value = 0 in Table 10 and 11). An increase of the weft density led to decrease the permanent (Figure 13) and total deformations responses and thus improved the dimensional stability and the comfort of the fabrics^61^. An increase of the platform temperature decreased the permanent deformation value of the 3D-PPOT materials (Figure 14).

**Table 9:** Permanent deformation of the 3D-PPOT materials (in µm): p-values and contributions of the main factors.

Factors	P-values	Contribution (%)
Platform temperature (°C)	0.00	6.97
Weft density (pck/cm)	0.00	50.7
Pattern	0.00	9.38

**Table 10:** Elastic deformation of the 3D-PPOT materials (in µm): p-values and contributions of the main factors.

Factors	P-values	Contribution (%)
Platform temperature (°C)	0.15	0.20
Weft density (pick/cm)	0.00	46.99
Pattern	0.59	0.03

**Table 11:** Total deformation of the 3D-PPOT materials (in µm): p-values and contributions of the main factors.

Factors	P-values	Contribution (%)
Platform temperature (°C)	0.005	0.92
Weft density (pick/cm)	0.00	44.07
Pattern	0.146	0.21

#### Theoretical models of the stress of the PLA printed layer

**Figure 15:**
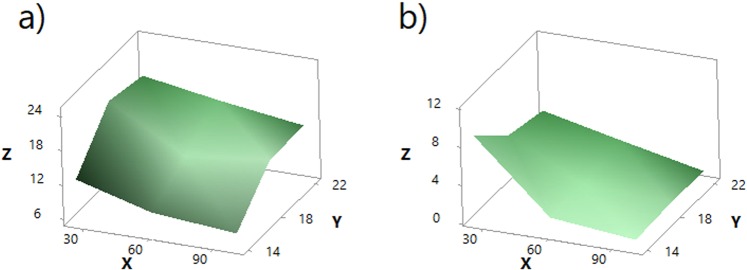
Theoretical models of stress of non-conductive PLA track (MPa) of 3D-PPOT materials in Z axis, in function of weft density (picks/inch) in Y axis and platform temperature (°C) in X axis in cross direction (**a**) using the statistical model of Eq. (3) and machine direction (**b**) using the statistical model of Eq. (4).

**Figure 16:**
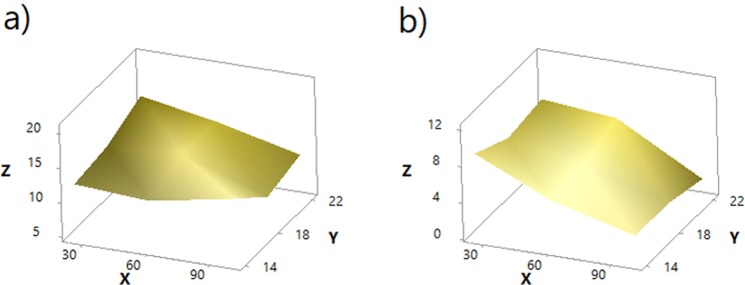
Theoretical models of stress of conductive PLA track (MPa) of 3D-PPOT conductive materials in Z axis, in function of weft density (picks/inch) in Y axis and platform temperature (°C) in X axis in cross direction (**a**) using the statistical model of Eq. (5) and machine direction (**b**) using the statistical model of Eq. (6).

Based on the previous findings, theoretical models of the stress of both conductive (Eqs (5) and (6)) and non-conductive (Eqs (3) and (4)) PLA printed layers of the 3D-PPOT materials were successfully (R-Square = [80–90%]) developed and their simulations presented in Figures 15 and 16.

Statistical models of stress of non-conductive PLA track:

- in cross direction
  3 $$Z=-\,9.1+2.97Y-0.32X-0.07{Y}^{2}+0.0014{X}^{2}+0.0042XY$$
- in machine direction
  4 $$Z=-\,1.8+1.95Y-0.32X-0.07{Y}^{2}+0.0014{X}^{2}+0.0042XY$$
  where Z is the stress of non-conductive PLA track, Y the weft density and X the platform temperature. The boundary conditions of X and Y are 14–22 pick/inch and 25–100 °C respectively.
  Statistical models of stress of conductive PLA track:
- in cross direction
  5 $$Z=8.18+0.356Y+0.0646X-0.00531XY$$
- in machine direction

6 $$Z=3.75+0.356Y+0.0213X-0.00531XY$$

where Z is the stress of non-conductive PLA track, Y the weft density and X the platform temperature. The boundary conditions of X and Y are 14–22 pick/inch and 25–100 °C respectively.

These theoretical models could support in enhancing the stress at rupture of the 3D-PPOT materials and most specifically the one of the PLA track by adjusting the platform temperature and the weft density of the fabric. It can also be noticed that the interaction between the factors weft density and platform temperature might be considered as its coefficient in the equation is significant.

#### Correlation between stress of the PLA printed track of 3D-PPOT materials and textile deformation prior to printing

**Figure 17:**
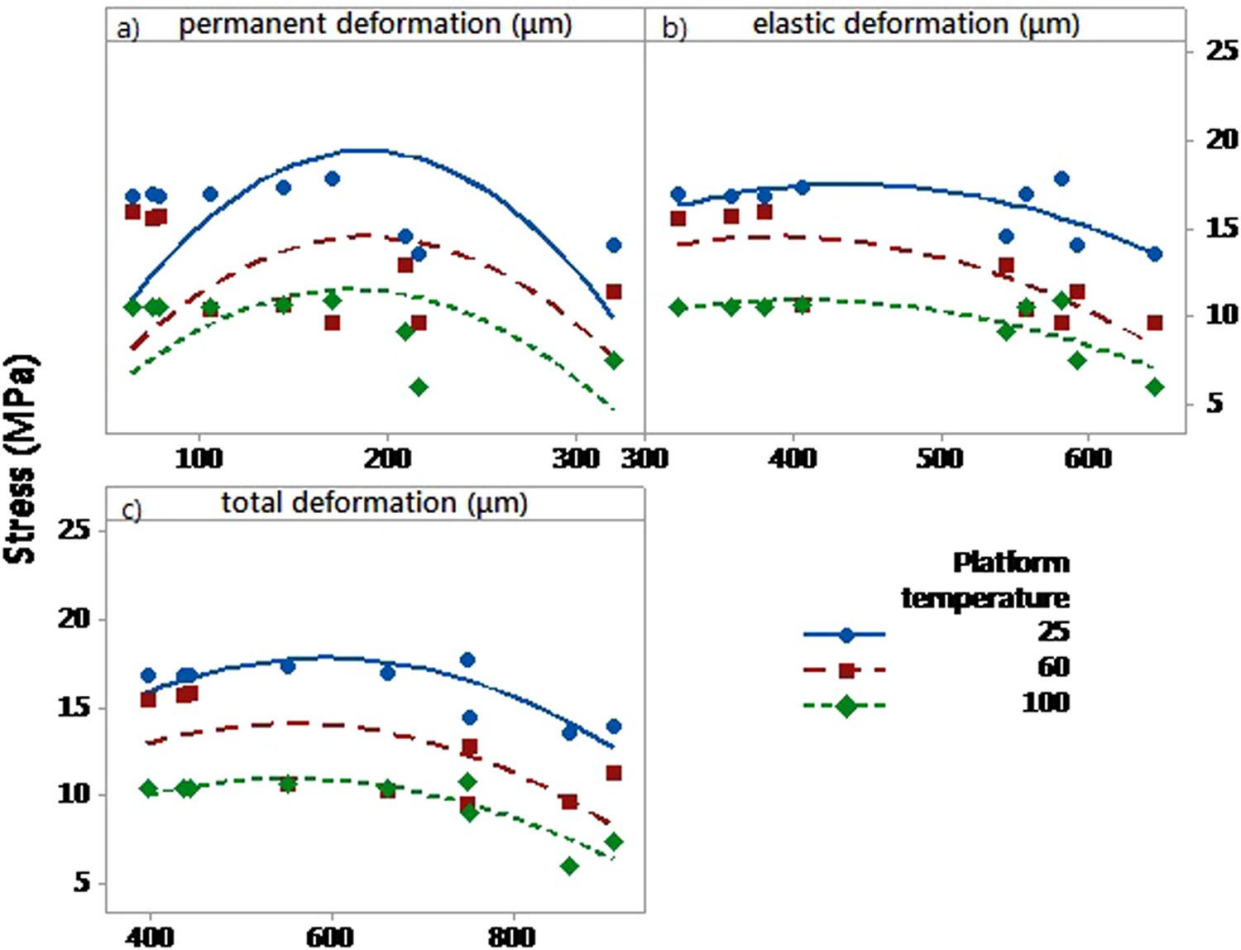
Experimental values and curve fits of the stress (MPa) of non-conductive PLA track of 3D-PPOT material printed at three platform temperatures 25, 60 and 100 °C in the cross direction on twill fabrics versus permanent (**a**), elastic (**b**) and total (**c**) deformations (in µm) of the fabrics before printing.

In order to demonstrate an existing correlation between the stress of the PLA printed layer of the 3D-PPOT materials and the deformations of the textile materials, simulated models were created (Eqs 7–12) based on the results and plotted in the cross direction (Figure 17). For each platform temperature, the stress of the PLA printed track presented a quadratic regression while increasing the permanent, elastic and total deformations. It means that deformation properties of the textile substrates could have a direct impact on the 3D-PPOT materials’ properties. With an increase of the permanent, elastic or total deformation of the textile, the stress of the non-conductive layer decreased to a minimum value and then remained stable. These results might be explained by an increase in adhesion due to higher penetration of the polymer through the textile in the case of higher permanent, elastic and total deformations. Higher adhesion could have led to lower stress of the polymeric track. Besides, low platform temperature resulted in decreasing the stress at rupture of non-conductive PLA track. It means that the optimization of the tensile strength of the 3D-PPOT materials might be done by considering the deformations of the textiles used as substrate and also the adjustment the platform temperature.

Statistical models of stress of non-conductive PLA track:

- in cross direction
  7 $${\rm{Z}}=28.2-9.0\times {10}^{-2}{{\rm{X}}}_{1}-0.25{\rm{Y}}-2.1\times {10}^{-4}{{{\rm{X}}}_{1}}^{2}+1.4\times {10}^{-3}{{\rm{Y}}}^{2}$$
  8 $${\rm{Z}}=27.41-1.3\times {10}^{-2}{{\rm{X}}}_{2}-0.25{\rm{Y}}-2.2\times {10}^{-5}{{{\rm{X}}}_{2}}^{2}+1.4\times {10}^{-3}{{\rm{Y}}}^{2}$$
  9 $${\rm{Z}}=31.98-3.2\times {10}^{-2}{{\rm{X}}}_{3}-0.25{\rm{Y}}-2.2\times {10}^{-5}{{{\rm{X}}}_{3}}^{2}+1.4\times {10}^{-3}{{\rm{Y}}}^{2}$$
- in machine direction

10 $${\rm{Z}}=11.13-2.0\times {10}^{-3}{{\rm{X}}}_{1}-0.25{\rm{Y}}-2.1\times {10}^{-4}{{{\rm{X}}}_{1}}^{2}+1.4\times {10}^{-3}{{\rm{Y}}}^{2}$$

11 $${\rm{Z}}=10.85+3.6\times {10}^{-3}{{\rm{X}}}_{2}-0.25{\rm{Y}}-2.2\times {10}^{-5}{{{\rm{X}}}_{2}}^{2}+1.4\times {10}^{-3}{{\rm{Y}}}^{2}$$

12 $${\rm{Z}}=12.39-9.3\times {10}^{-3}{{\rm{X}}}_{3}-0.25{\rm{Y}}-2.2\times {10}^{-5}{{{\rm{X}}}_{3}}^{2}+1.4\times {10}^{-3}{{\rm{Y}}}^{2}$$

where Z is the stress of non-conductive PLA track, X_1,_ X2 and X_3_ are the permanent, elastic and total deformations of the textile before printing and Y the platform temperature. The boundary conditions X_1,_ X2 and X_3_ are 50–350 mm, 200–700 mm and 350–950 mm respectively.

The R-squares of the different models are 84.2%, 82.7% and 81.6% for the combination [Eqs (7) and (10)], [Eqs (8), (9), (11) and (12)] respectively. Similar models could be developed for the stress of the track of conductive 3D-PPOT materials.

#### Wash durability of the 3D-PPOT materials

**Figure 18:**
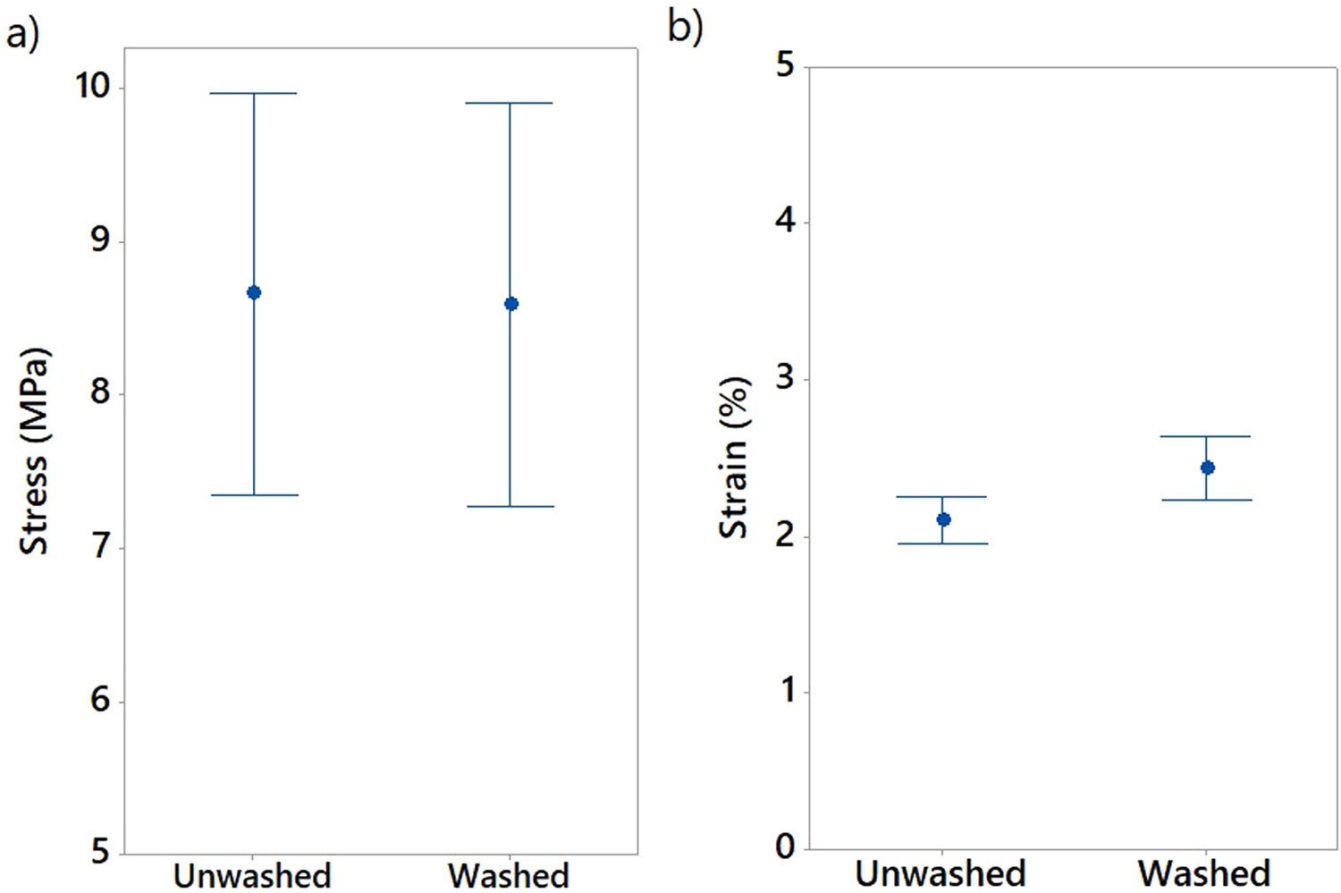
Stress in MPa (**a**) and Strain in % (**b**) at break of non-conductive PLA track of 3D-PPOT materials before and after washing process.

**Figure 19:**
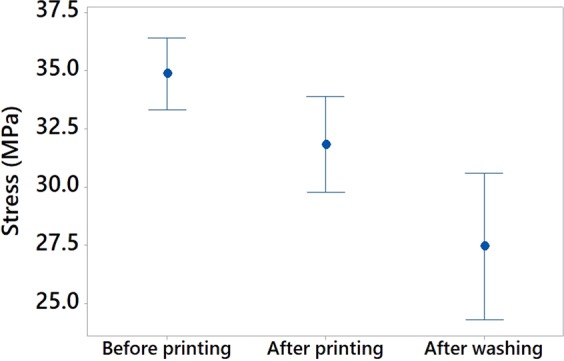
Stress (in MPa) of PET woven fabric before and after printing using non-conductive PLA filament (3D-PPOT materials) and after washing of 3D-PPOT materials.

**Figure 20:**
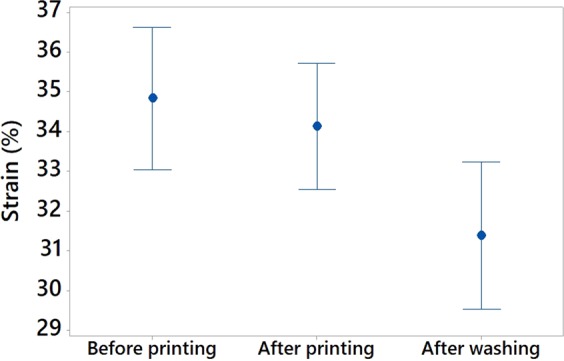
Strain (in %) of PET woven fabric before and after printing using non-conductive PLA filament (3D-PPOT materials) and after washing of 3D-PPOT materials.

**Figure 21:**
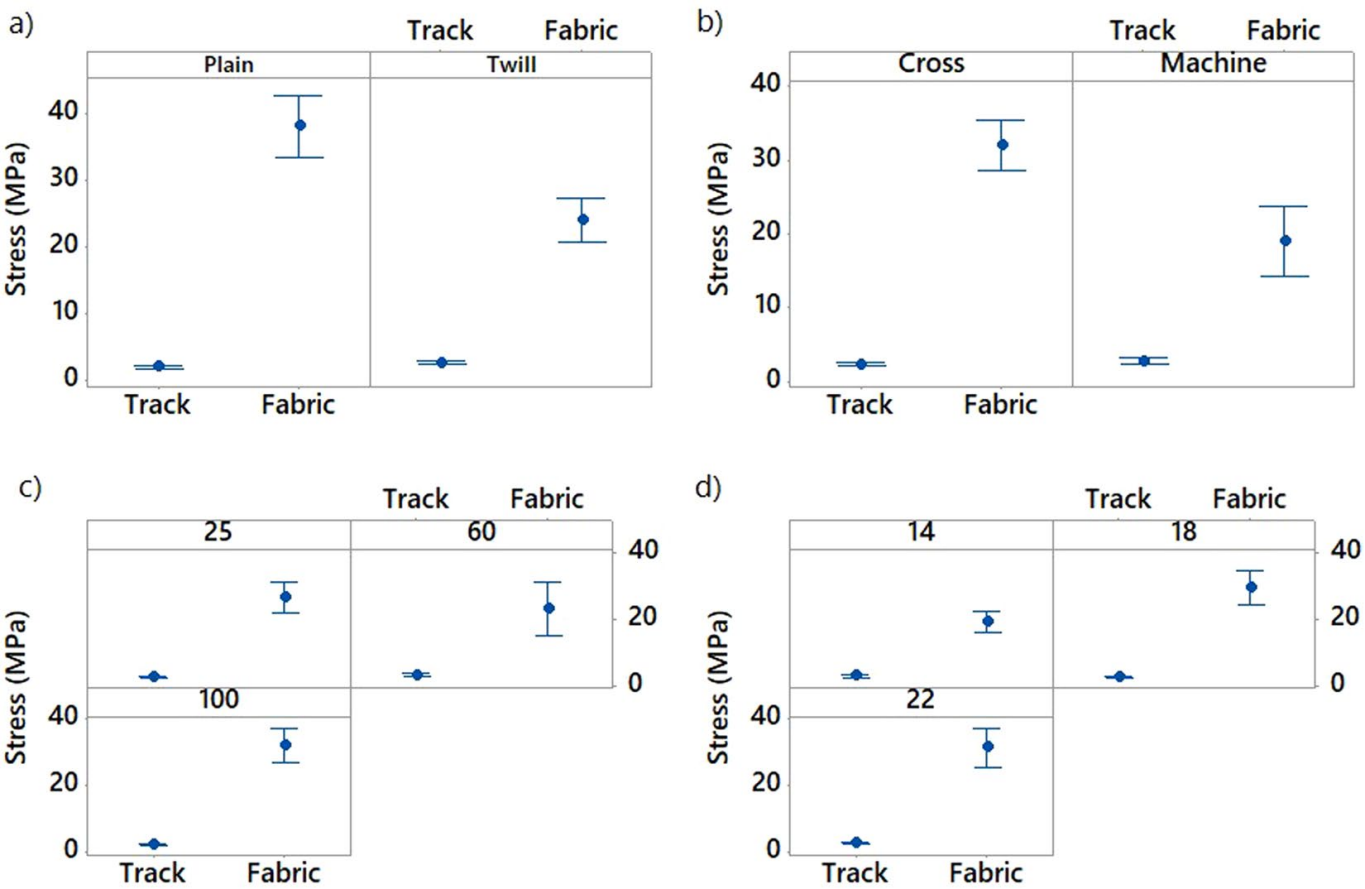
Effect of textile pattern (**a**), textile orientation (**b**), platform temperature (**c**) and textile weft density (**d**) on stress (MPa) after washing test (wash durability) of conductive 3D-PPOT materials made of conductive PLA track and PET fabric. Both the stress of the PLA track and PET fabrics have to be considered.

In order to assess the durability of the 3D-PPOT materials using non-conductive PLA filament deposited on PET woven fabrics, their stress and strain after one cycle of washing process, at 40 °C during 15 minutes using ECE Formulation Non-Phosphate Reference detergent, were measured and their mean value and interval reported (Figure 18(a,b)). As a whole, the washing process did not impact the stress and strain of the PLA track. However, the stress and the strain of the PET fabric of the 3D-PPOT material decreased due to the damage to some fibers structure after washing (Figures 19 and 20). Similarly to the results before washing process, the direction and weft density of the textile substrates and the platform temperature were the factors impacting the stress of the PLA track stress (Figure 21) the most but its strain was not influenced by any factors.

## Conclusions

In the present work, the effect of the textiles’ properties and build platform temperature on the tensile and deformation of deposited virgin or conductive PLA printed onto polyethylene terephthalate (PET) woven fabrics (3D-PPOT materials) were investigated. The wash durability and the influence of conductive fillers in PLA were also approached in this study.

Based on the findings, the build platform temperature of the printer, the fabric orientation and the weft density of the PET woven fabrics had a significant influence on the tensile and deformation properties of the 3D-PPOT materials. When analyzing the overall tensile properties of 3D-PPOT materials, it was found that the ranges of stress and strain at rupture were approximately three times lower for the non-conductive and the conductive PLA track compared to those of the PET fabric due to the low flexibility of the printed track and weak adhesion to the textile substrate. It might be explained by the low flexibility of the printed track and weak adhesion to the textile substrate. Thus, an improvement of the adhesion between the layers could improve the tensile properties of the PLA printed layer and thus, the one of the 3D-PPOT materials. Besides, an increase of the platform temperature increased the crystallization rate of the conductive and non-conductive PLA filaments and thus, decreased the tensile strength of PLA printed track of the 3D-PPOT materials. Also, printing in the cross direction demonstrated better stress at rupture due to better affinity with the PET monofilament used as weft yarn compared to the one with the PET twisted yarn used as warp yarn. Based on the findings, the tensile resistance of the non-conductive PLA printed track presented a good durability after washing process or after incorporating conductive fillers, however, the stress at rupture of the woven fabric (after printing process) was impacted by the washing process.

The elastic, total and permanent deformations of the 3D-PPOT materials were lower than the one of the fabric before polymer deposition which demonstrated a better dimensional stability and higher stiffness of the materials when using 3D printing deposition process. The higher the permanent, elastic and total deformations of the PET textile substrate presented a quadratic effect on the tensile of the PLA printed track.

The findings are important in the development of smart textiles using deposition process as it supports the optimization of their mechanical resistance, durability and comfort. Better tensile properties could be obtained by using more flexible filaments and improving the adhesion between the layers.

## Supplementary information

Dataset 1

## Data Availability

The authors declare that there is no data available for this manuscript.
