# Supplementary material for: Stress, strain and deformation of poly-lactic acid filament deposited onto polyethylene terephthalate woven fabric through 3D printing process
Source: Sci Rep. 2019 Oct 4;9:14333. doi: 10.1038/s41598-019-50832-7 (PMC6778127; doi:10.1038/s41598-019-50832-7)
Supplement: Supplementary file 1 — Dataset 1 [file 41598_2019_50832_MOESM1_ESM.pdf]

Stress, strain and deformation of poly-lactic acid filament deposited onto polyethylene terephthalate woven fabric through 3D printing process

Prisca Aude Eutionnat-Diffo ·
Yan Chen · Jinping Guan ·
Aurelie Cayla · Christine Campagne ·
Xianyi Zeng · Vincent Nierstrasz

Corresponding author: Prisca Aude Eutionnat-Diffo
Textile Materials Technology, Department of Textile Technology, Faculty of Textiles, Engineering and Business, University of Borås, Borås, 50190, Sweden
Laboratoire de Génie et Matériaux Textiles, ENSAIT/GEMTEX, Lille, 59000, France
College of Textile and Clothing Engineering, Soochow University, Suzhou, 215006, Jiangsu, China
Correspondance to: E-mail: prisca.eutionnat-diffo@ensait.fr/ prisca.eutionnat@hb.se

Yan Chen
College of Textile and Clothing Engineering, Soochow University, Suzhou, 215006, Jiangsu, China
E-mail: yanchen@suda.edu.cn

Jinping Guan
College of Textile and Clothing Engineering, Soochow University, Suzhou, 215006, Jiangsu, China
E-mail: guanjinping@suda.edu.cn

Aurelie Cayla
Laboratoire de Génie et Matériaux Textiles, ENSAIT/GEMTEX, 59000, Lille, France
Email: aurelie.cayla@ensait.fr

Christine Campagne
Laboratoire de Génie et Matériaux Textiles, ENSAIT/GEMTEX, 59000, Lille, France
E-mail: christine.campagne@ensait.fr

Xianyi Zeng
Laboratoire de Génie et Matériaux Textiles, ENSAIT/GEMTEX, 59000, Lille, France
Email: xianyi.zeng@ensait.fr

Vincent Nierstrasz
Textile Materials Technology, Department of Textile Technology, Faculty of Textiles, Engineering and Business, University of Borås, Borås, 50190, Sweden
E-mail: vincent.nierstrasz@hb.se

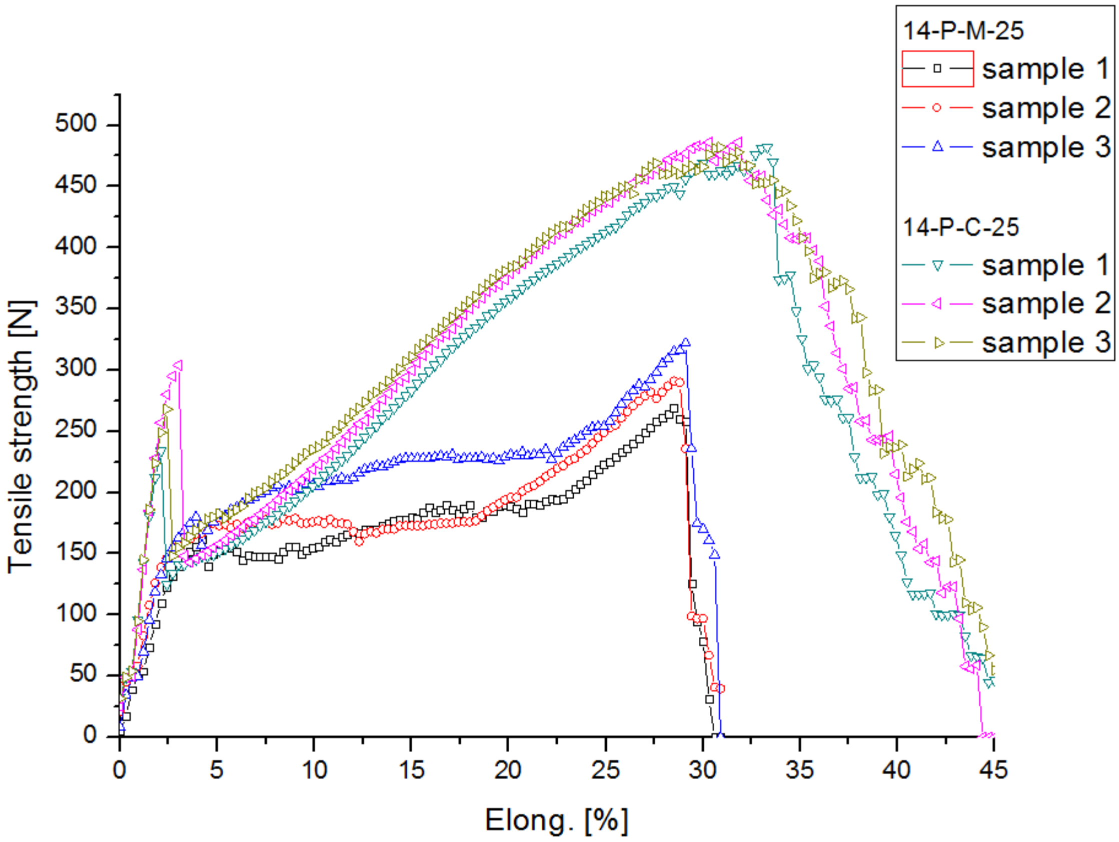

Fig. 1 Tensile force–Elongation curves (three replicates) of 3D-PPOT using PLA filament printed on 14picks/inch PET woven fabric at 25◦C in cross direction (14-P-C-25) and machine direction (14-P-M-25).
